# Age Assessment through Root Lengths of Mandibular Second and Third Permanent Molars Using Machine Learning and Artificial Neural Networks

**DOI:** 10.3390/jimaging9020033

**Published:** 2023-02-01

**Authors:** Vathsala Patil, Janhavi Saxena, Ravindranath Vineetha, Rahul Paul, Dasharathraj K. Shetty, Sonali Sharma, Komal Smriti, Deepak Kumar Singhal, Nithesh Naik

**Affiliations:** 1 Department of Oral Medicine and Radiology, Manipal College of Dental Sciences, Manipal, Manipal Academy of Higher Education, Manipal 576104, Karnataka, India; 2 Department of Radiation Oncology, Massachusetts General Hospital, Harvard Medical School, Boston, MA 02115, USA; 3 Department of Data Science and Computer Applications, Manipal Institute of Technology, Manipal Academy of Higher Education, Manipal 576104, Karnataka, India; 4 Department of Biomedical Dental Sciences, College of Dentistry, Imam Abdulrahman Bin Faisal University, Dammam 34212, Saudi Arabia; 5 Department of Public Health Dentistry, Manipal College of Dental Sciences, Manipal Academy of Higher Education, Manipal 576104, Karnataka, India; 6 Department of Mechanical and Industrial Engineering, Manipal Institute of Technology, Manipal Academy of Higher Education, Manipal 576104, Karnataka, India; 7 Curiouz TechLab Private Limited, BIRAC-BioNEST, Manipal Government of Karnataka Bioincubator, Manipal 576104, Karnataka, India

**Keywords:** age estimation, artificial intelligence, Deep Learning, root length, forensic odontology, Machine Learning

## Abstract

The present study explores the efficacy of Machine Learning and Artificial Neural Networks in age assessment using the root length of the second and third molar teeth. A dataset of 1000 panoramic radiographs with intact second and third molars ranging from 12 to 25 years was archived. The length of the mesial and distal roots was measured using ImageJ software. The dataset was classified in three ways based on the age distribution: 2–Class, 3–Class, and 5–Class. We used Support Vector Machine (SVM), Random Forest (RF), and Logistic Regression models to train, test, and analyze the root length measurements. The mesial root of the third molar on the right side was a good predictor of age. The SVM showed the highest accuracy of 86.4% for 2–class, 66% for 3–class, and 42.8% for 5–Class. The RF showed the highest accuracy of 47.6% for 5–Class. Overall the present study demonstrated that the Deep Learning model (fully connected model) performed better than the Machine Learning models, and the mesial root length of the right third molar was a good predictor of age. Additionally, a combination of different root lengths could be informative while building a Machine Learning model.

## 1. Introduction

Age estimation plays a remarkable role in forensic medicine. From identifying individuals and casualties in natural disasters to decrypting medico-legal cases, it effectively assists in narrowing search possibilities [1,2,3]. Age estimation methods also help to assess instances of legal maturity in judging prosecution as a juvenile or as an adult, which aids in deciding the severity of punishment for criminal offenses. Teeth are preferred for age estimation as they have high durability and resistance to heat, chemicals, putrefaction, and other factors. Many fields, such as anthropology, archaeology, forensic science, pediatric dentistry, and orthodontics, use developing teeth to measure and estimate age and maturity [4,5].

Age estimation using teeth can be performed through visual, radiographic, chemical, and histological methods [5]. Radiographic methods are based on estimating the stages of dental development by identifying the mineralization of the crown and root apex maturation [6]. The visual technique is based on the eruption order of teeth and morphological indicators of aging, such as attrition, deposition of secondary dentin, and color changes. The histological methods require the extraction/sectioning of the tooth, whereas the chemical examination of dental hard tissues determines changes in ion levels with age [7]. Morphological features, such as the amount of occlusal attrition, coronary secondary dentin deposition, cementum apposition on the root apex, root resorption, and dentinal transparency are used in Gustafson’s age estimation method [8]. Similarly, Moore, Fanning, and Hunt have studied 14 stages of mineralization of developing teeth using panoramic and lateral oblique radiographs. Kvaal et al. and Cameriere et al. have used pulp size measurements through intra–oral periapical radiographs [9,10,11].

The late adolescence to early adulthood transition phase has vital implications in medico–legal cases. Nearly all permanent teeth would have completed their eruption process at this age except for the third molars. Hence, this transition age primarily depends on the chronology of the mineralization of third molars [12]. However, in most conditions, third molars are either congenitally missing, impacted, extracted, or malpositioned, making them less suitable for age assessment [13]. Previous studies have determined the second molar’s maturation stages as a valid marker for age assessment. Hence, in this transition phase of adolescence to early adulthood, using the third and second molar can prove beneficial in age estimation [8]. Although earlier studies have employed the subjective analysis of root forms and compared them with compiled charts, they were associated with subjective bias [9,10,11,14]. To overcome these subjective errors, observer–independent and objective methods are essential. Measurements allow for the development of mathematical models and parametric statistics. Root length measurements in age estimation in permanent teeth can eliminate methodological variations.

An Artificial Neural Network (ANN) uses mathematical models and algorithms to analyze and interpret data. Machine Learning (ML) is a type of data analysis that “learns” intrinsic statistical patterns to make predictions on unseen data. Deep Learning is a Machine Learning technique that employs multi–layer mathematical operations to learn and infer complicated inputs, such as images [15]. Neural networks rely on training data to learn and improve their accuracy over time. Once these learning algorithms are tuned for precision, they are powerful tools in artificial intelligence, allowing for classifying and clustering data at a high velocity. Neural networks were limited by computing power. However, advancements in Big Data analytics and access to higher computing resources have permitted more extensive, more sophisticated neural networks. Deep Learning is a subset of Machine Learning that aids image classification, language translation, and speech recognition. It can solve any pattern recognition problem without human intervention [16,17]. Tasks in speech recognition or image recognition can take minutes rather than hours compared with manual identification by human experts [18]. Deep Learning’s application in forensic medicine has been explored over recent years due to its advantages of accuracy and precision in age and gender estimation [18,19,20]. 

A previous study utilized X–ray images of teeth along with Machine Learning techniques to achieve 97% accuracy in age estimation, which implies that Machine Learning can be applied effectively in forensic investigations to obtain accurate and quick results [19]. Gender determination on panoramic radiographs using neural networks also exhibited good gender prediction compared with other methods, such as logistic and discriminant analysis [20]. Age estimation using artificial intelligence through first molar images of both the right and left sides of the maxilla and mandible has also yielded highly accurate results [21]. Deep Convolutional Neural Networks using orthopantomography have also been applied to estimate the age of children using the features of teeth [21]. Hence, the present study is carried out to explore the efficacy of Machine Learning and Deep Learning in age estimation using the second and third molar root lengths. Clinical judgments with the help of Machine Learning models in the health care system using interpretable and precise models are beneficial and in demand. Hence, using the above background, the present study was planned to explore the use of machine learning. 

## 2. Materials and Methods

### 2.1. Study Design

This retrospective study was carried out from the archives of the Oral and Maxillofacial Radiology section (from March 2017 to March 2021) at Manipal College of Dental Sciences, Manipal. We selected 1000 digital panoramic radiographs of individuals aged between 12 and 25 years. Radiographs of individuals belonging to the southern part of the state (South Indian population) were considered after verifying their address from medical records. The difference between the date of birth provided in the dental record and the date on which the radiograph was taken was considered to calculate the age of the individual. Radiographs with diagnostically acceptable images of intact mandibular second and third molars were included in the study. Radiographs with the third and second molars missing or obscured due to artifacts, trauma, or fracture lines of the mandible passing through these molars were excluded. The radiographs that showed various lesions, syndromes, and developmental disorders were also excluded.

The study was conducted after receiving approval from the Institutional Ethics Committee (I.E.C. No: 249/2021).

### 2.2. Measurements

**Figure 1:**
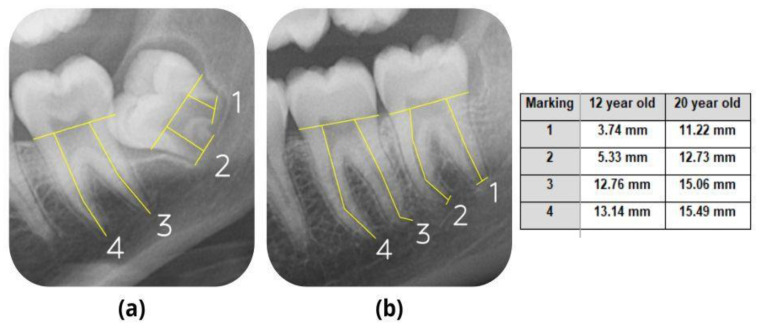
Measurement of mesial and distal root lengths of left mandibular second and third molars in (**a**) 12–years–old female patient and (**b**) 20–years–old male patient.

The lengths of the roots of the right and left mandibular second and third molars were measured using ImageJ, a Java–based image processing software developed at the National Institute of Health and the Laboratory for Optical and Computational Instrumentation. (Figure 1). A scale for measurement was set using a fixed distance in pixels and a known distance in millimeters (mm). The length of the root was measured by dividing the crown and root portion by a horizontal line passing through the cement–enamel junction (C.E.J.) on the mesial and distal portion of the crown.

Measurements were made by drawing a vertical line from this dividing line to the visible apex of the root, as shown in Figure 1. Mesio–buccal and distobuccal root lengths of both the right and left second and third mandibular molars were measured (Figure 1) and tabulated on an M.S. Excel spreadsheet along with the age and gender of the individual. All the measurements were made by a trained dental graduate (Observer 1—J.S.). The measurements were made after reaching a consensus with two oral radiologists (V.P. and R.V.) and a trained dental graduate (J.S.). Intra–observer reliability was derived by repeating the measurements of ten percent of the sample size by Observer 1 (J.S.) on a different day. The intra–observer correlation coefficient was calculated to assess the agreement, and it was found to be in very good agreement, with a value of 0.96.

### 2.3. Data Processing

**Figure 2:**
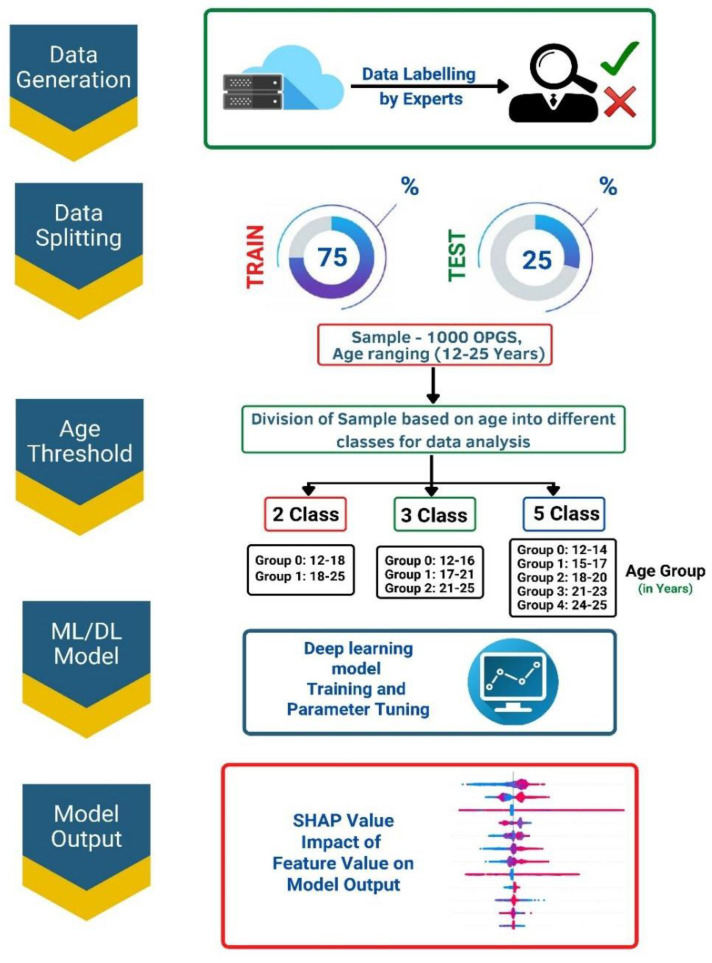
Block diagram illustrating the various steps involved in building a Deep Learning–based tool for automated analysis and classification of data into the specified categories.

The output was classified into three categories, namely 2–Class, 3–Class, and 5–Class, depending on the age distribution, as shown in Figure 2. The dataset included 1000 patients, with information on distal and mesial root lengths from second and third molars on the left and right sides.

We used 75% of the data for training and 25% for testing. Missing data imputation was conducted by replacing the empty spaces with the mean of that particular column wherever data were missing. The data were normalized by making the observed values’ mean and standard deviation 0 and 1, respectively. We also used Linear Discriminant Analysis (LDA) as the feature extractor as an alternative to the strategy with no feature selection in building a Deep Learning model.

### 2.4. Computational Techniques

Our research utilized explanatory methods, such as SHAP, which aids in understanding ML model prediction [22], and we also used Support Vector Machine (SVM), Random Forest (RF), and Logistic Regression (LR) algorithms for classification, training, testing, and analyzing the data [23,24,25]. Different regressors were used to compute the predicted value versus the true value using a Random Forest (RF) Regressor; Extra Tree Regression (ETR); XGBoost Regressor, which is a decision tree–based ensemble Machine Learning algorithm; and a Gradient Boosting Regressor [26,27,28,29].

Linear Discriminant Analysis (LDA) is a feature extraction strategy that uses knowledge from all classes to create a new axis to project data in such a way that the intra–class variance is significantly reduced while the inter–class average distance is enhanced. As an alternative to the strategy with no feature selection, we also used LDA as the feature extractor.

The Deep Learning model is used to obtain various outputs and compare their results to fine–tune the considered models. The ideal and optimum neural network design in this study was determined using AutoKeras, an autoML platform built on the Keras framework. A two–layer fully connected network was constructed by Autokeras (layer 1: 32 neurons; layer 2: 16 neurons), which was then succeeded by a final classification layer with just 1 neuron and a sigmoid function. To reduce overfitting, a dropout layer (0.2) was inserted after layer 2. The network was trained using binary cross–entropy loss with a batch size of 16. The Deep Learning model was trained by splitting the training data (75% of the original dataset) further into training data for DL (75%) and validation data for DL (25%).

### 2.5. Performance Measurement

A confusion matrix is useful in visualizing the predictive performance of a Machine Learning model with respect to the actual labels. Precision and recall can be computed directly from the confusion matrix. Hence, Accuracy, AUC, Recall, and Precision were used to evaluate the developed model. The commonly used diagnostic evaluation tool is the AUC–ROC (area under the curve–receiver operating characteristic) score. It is a measure of how effectively a model can distinguish between classes. The true positive rate (sensitivity) is plotted as a function of the false positive rate (100 specificity) at various threshold settings, and the area the curve covered is called the AUC–ROC score. AUC has a value ranging from 0 and 1. A model with 100% false predictions has an AUC of 0.0, while a model with 100% accurate predictions has an AUC of 1.0. Statistical significance of the improvement in AUC between different methods and classifiers was calculated using standard error (SE) and a 2-tailed *p*-value of 0.05 [30,31].

The regression model performance was assessed using mean absolute error (MAE), root mean square error (RMSE), and R square, which are critical to evaluate the performance of any regression model. RMSE and MAE measure the distance between real and predicted value, hence the model predictiveness increases with decreasing RMSE and MAE. Pearson’s correlation was used to find the correlation between the root length and patient age. This method is used to analyze whether a strong relationship exists between the dependent and independent variables. Hence, the correlation coefficient r is used to measure the strength of the relationship among various variables. This analytical technique is based on the premise that determining the significance of a pertinent attribute in the data can be conducted by analyzing the strength of the association between dependent and target variables [32,33,34,35].

### 2.6. Feature Importance

In the present study, the Shapley Additive Explanations, or SHAP technique, is used to analyze each feature’s value affecting the anticipated output to comprehend the suggested classification models. SHAP was created based on Shapley’s values. As a concept for a cooperative game theory solution, it was initially presented by Lloyd Shapley in 1951. SHAP analyzes each feature and its importance to the model output based on Shapley data. In the SHAP summary plot, the X and Y axes, respectively, depict the SHAP and feature values, and a color map is used to indicate the SHAP values of each feature (blue and red illustrate low and high tooth length values, respectively [36,37].

## 3. Results

**Figure 3:**
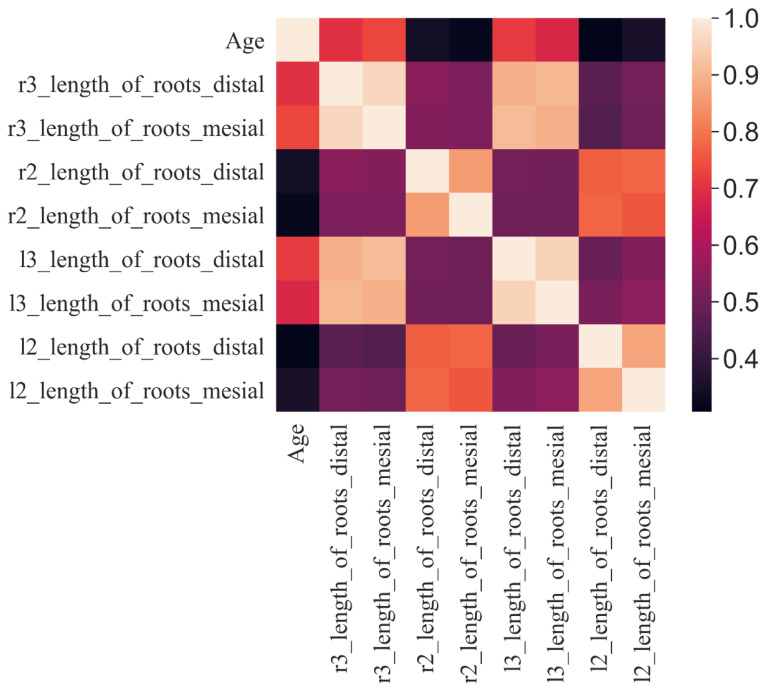
Heatmap representation of Pearson correlation among age groups and root lengths.

Figure 3 displays correlations among the demographic factor (age) and clinical parameters (root length). The correlation plot showed limited correlation between age and root length. The left and right third molar mesial and distal teeth showed a moderate (0.7) correlation with age.

The classification performance of the established algorithms is described in Table 1 and Table 2, respectively. Table 1 shows the classification performance analysis of the LDA with the feature extractor. The SVM demonstrates the highest accuracy of 86.8%, 66%, and 44% in 2–Class, 3–Class, and 5–Class, respectively, whereas it is observed that the accuracy is lesser in the RF classifier. This output is due to the denser network of SVMs compared with the RF. The Recall of the SVM is to be the best in comparison with all classes and classifiers, making it the most suitable. When compared with the SVM, RF had the highest AUC (0.83). However, across all employed classifiers, the AUC improvement is statistically significant.

Table 2 shows the classification performance analysis of the LDA with no feature extractor. The SVM showed the highest Accuracy of 86.4% and 66% in 2–Class and 3–Class, respectively, whereas an Accuracy of 42.8% was obtained for 5–Class. The RF showed the highest Accuracy of 47.6% for 5–Class.

The SVM had the highest Accuracy of all three; however, the Precision of the RF in 3–Class made it the best performer for 3–Class, with the SVM regaining the best precision in 5–Class. Hence, the specific models can be used in the following class distributions: 2–Class—SVM; 3–Class—RF; and 5–Class—SVM.

The Deep Learning classification (Table 3) shows the highest Accuracy, AUC, and Recall in comparison with the other Machine Learning models considered in the study.

**Figure 4:**
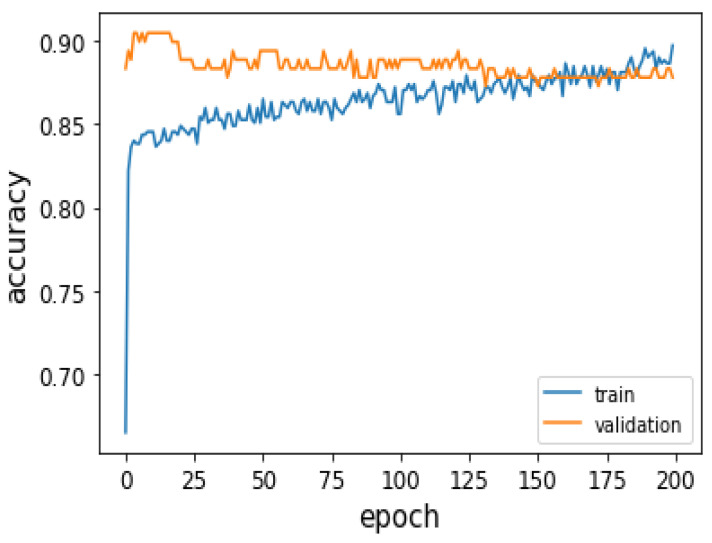
Accuracy plot of training and validation of Deep Learning Neural network model.

Figure 4 is a plot showing the Accuracy of the Deep Learning network training and the validation. It is observed that the Accuracy increases with an increase in epochs with respect to the training dataset, and the validation Accuracy changes from 94% to 87.2%. Hence, the training and validation Accuracy is balanced, indicating that the network is not of high bias or variance.

**Figure 5:**
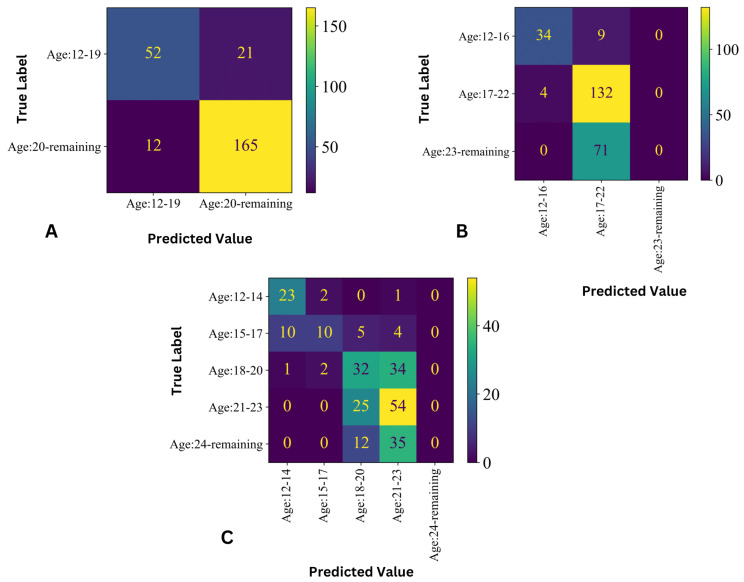
Confusion matrix from the best predictive model: (**A**) 2–Class prediction, (**B**) 3–Class prediction SVM, and (**C**) Random Forest 5–Class prediction.

The confusion matrix from the best predictive model of the 2–Class, 3–Class, and 5–Class models is shown in Figure 5. Figure 5A illustrates the confusion matrix of the best 2–Class model, where the misclassification rate of the samples from the age 20–25 subjects (Group 1) is very low compared with ages 12–19 (Group 0). The confusion matrix from the best 3–Class predictive model is displayed in Figure 5B, which shows that all samples from age 23 and above (Group 2) were misclassified. As the samples were grouped with a three–year difference, the 5–Class confusion matrix (Figure 5C) became more diverse. There is a good amount of false positive and false negative samples in the age groups 18–20 (Group 2) and 21–23 (Group 3).

**Figure 6:**
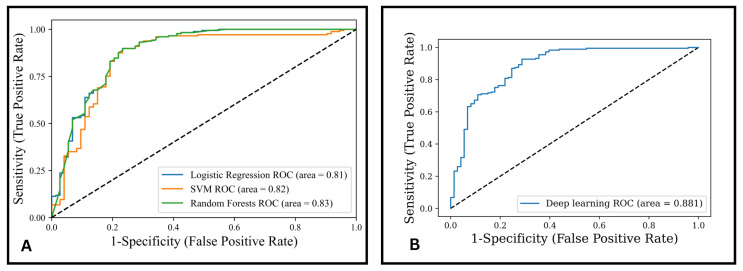
The diagnostic evaluation of the model for 2–Class prediction of (**A**) ROC curve for the LR, SVM, and RF, and (**B**) ROC curve for the DL algorithm.

Figure 6 depicts the ROC curve from the best Machine Learning and Deep Learning models for two–class classification. The Deep Learning model’s AUC (0.88) improvement was compared with the best model from the LDA feature selector (RF: 0.83 AUC) and no feature selector (SVM: 0.82 AUC). The Deep Learning model was not statistically significant at *p* = 0.05 compared with LDA (*p* = 0.129) and no feature selector (*p* = 0.074).

**Figure 7:**
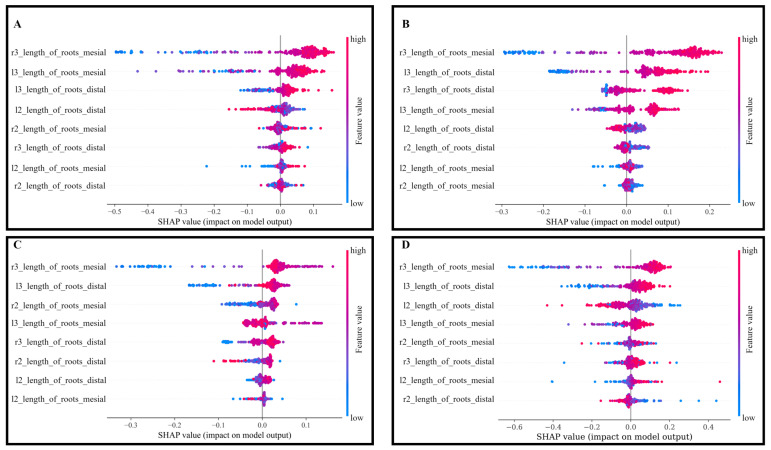
SHAP plots showing feature importance in descending order by bee swarm plot: (**A**) 2–Class classification (SVM): best result; (**B**) 2–Class classification (Random Forest): second best result; (**C**) 3–Class classification (SVM): best result; and (**D**) 2–Class classification (Deep Learning).

As shown in Table 2, the SVM gives the highest accuracy in all the classes, except Deep Learning in 2–Class. SHAP was used to assess the best Machine Learning and Deep Learning model’s predictive performance (shown in Figure 7). Figure 7a shows the SHAP value with respect to the length of the mesial and distal roots. In the case of 2–Class, RF performance is the second best, which is observed in comparison with the smaller number of positive SHAP values (Figure 7b) using the SVM. In the case of the 3–Class classification, as shown in Figure 7c, the SVM classifier provides the best result. The SHAP plot (Figure 7d) from the Deep Learning model showed the right side third molar mesial root as the top distinguishable feature.

## 4. Regression

**Figure 8:**
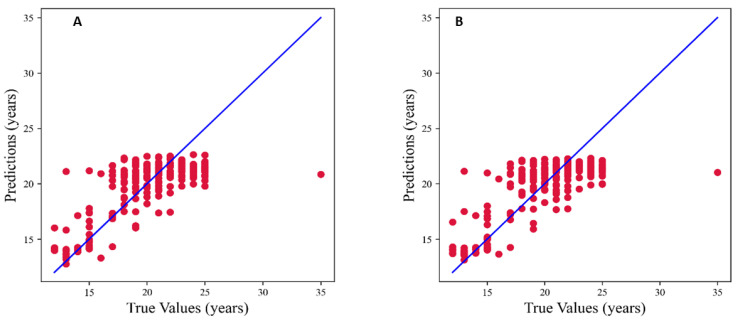
Plot of prediction versus true value: (**A**) Random Forest Regressor and (**B**) Extra Tree Regressor.

Figure 8a shows the output of the RF Regressor, and the plot shows the data points are more focused on the mid–R square value. Figure 8b shows that the Extra Tree Regressor has a more scattered plot, with the R square value going down.

Table 4 shows the results obtained from various regressors used in the present study with R square values ranging from 0.56 to 0.58.

Random Forest Regressor and Extra Tree Regressor models had an R square value of 0.58; however, the Extra Tree Regressor generated lower MAE and RMSE scores.

## 5. Discussion

Teeth are a reliable adjunct in age estimation as they are easily obtained as evidence even after other body parts have disintegrated. The development of human dentition exhibits a chronological pattern, with crown formation to root completion exhibiting sequential calcification. They further follow age–wise eruption and exfoliation patterns. Previous studies in the literature have used these factors for estimating age [5,6,7,8]. However, the length of the root as an indicator of age has not been explored to date.

The roots of the second and third permanent molars are the only dental structure that continues their development throughout adolescence after all the other teeth have erupted [12,13,38]. The root formation of the second permanent molar completes at the age of 14–16 years, while the root of the third permanent molar continues to grow even after the complete development of the second permanent molar [39,40]. This pattern of growth makes them a viable tool for age estimation. Although a few studies are using the morphological features of second and third permanent molars for the prediction of age [12,39,40,41], no studies have used the root length in age estimation. Hence, in the present study, we evaluated the efficacy of root length in the age assessment amongst individuals in the transition phase of early childhood to late adolescence. Haaviko et al. [42] developed an age estimation method based on the recognition of 12 radiographic stages of 4 teeth. Wilmott et al. [43] employed Haaviko’s method and found that the stage wise assessment of root formation estimated age more accurately than eruption level. Maber M et al. [38] stated that the second molar showed higher accuracy, while Mesotten et al. [44] and Gunst K et al. [41] reported third molar root formation as an appropriate indicator of age estimation [41,44]. Hence these patterns of accuracy determined by these researchers justify the usages of mandibular second and third permanent molars.

When the sample of root lengths was divided as per the age into different groups, the classification with two groups (Class 2 with Group 0 having 12–18 years and Group 1 having 19–25 years) showed the highest accuracy of 86.8% (SVM), 86.0% (RF), and 84.8% (LR). Group 0 with ages ranging from 12 to 18 years had 0.29 percent of patients misclassified, whereas Group 1, which has ages from 19 years and above, had only 0.07 percent of patients misidentified, indicating that Group 0 teeth lengths could be close to Group 1. Group 0 showed more variation in root lengths compared with Group 1. This could be attributed to the fact that a majority of root formation occurs from 12 to 18 years of age. 

The three–group classification (Class 3 with Group 0 having 12–16 years, Group 1 having 17–21 years, and Group 2 having 22–25 years) depicted an accuracy of 66.0% (SVM), 60.0% (RF), and 60.4% (LR). The five–group classification (Class 5 with Group 0 having 12–14 years, Group 1 having 15–17 years, Group 2 having 18–20 years, Group 3 having 21–23 years, and Group 4 having 24–25 years) showed an accuracy of 44.0% (SVM), 42.4% (RF), and 40.4% (LR). This showed that when the sample was divided into multiple smaller age groups, decreased accuracy was noted. This is because in Machine Learning, most of the Group 2 samples were predicted to be Group 1 due to the greater fluctuation of the root length below the age of 21 years. Therefore, the Machine Learning classifier was unable to differentiate much between the smaller classification—Class 5 in comparison with Class 3 and Class 2—resulting in a reduction in classification performance. However, the poor performance for 5–Class classification does not inherently imply that the proposed classification strategy is insufficient; it could be the consequence of the limited sample size in each group when dividing the data into five classes.

The correlation plot showed a low–to–moderate correlation (0.7) among the root lengths. Hence, combining different root lengths in age estimation can be more beneficial while building Machine Learning models. The SVM showed the highest accuracy, which can be attributed to the denser network of the SVM compared with the RF. The recall value of the SVM is also best in comparison with all classes. Linear Discriminant Analysis (LDA) is a feature extraction strategy that uses knowledge from all classes to create a new axis to project data in such a way that the intra–class variance is significantly reduced while the inter–class average distance is enhanced [32,33]. The incorporation of the LDA feature–extracting technique to find the subset of features of the data has proven to be efficient and has resulted in an increase in classification accuracy of 10%. LDA feature selection was compared with the “no feature selector” approach. It was observed that the accuracy decreased if there was no feature extractor. Hence, the LDA extractor was the preferred method [24,25,27]. The SVM had the highest accuracy of all three; however, the precision of the RF in 3–Class made it the best performer for 3–Class, with the SVM regaining the best precision in 5–Class. This shows that the specific models (2–Class: SVM; 3–Class: RF; and 5–Class: SVM) can be used in the following class distributions.

SHAP was used to explain the Machine Learning models [32]. SHAP analysis offered two significant benefits. SHAP provided the knowledge of which features had the strongest influences on the multiclass classifier model. Second, SHAP offered an explainability for the black box Machine Learning and Deep Learning models, aiding in the development of confidence and acceptability for these models. It is important to note that the patterns demonstrated in the SHAP values depicted the trend learned by the Machine Learning model rather than the actual features themselves [32].

In the present study, it was evident that the right third molar–mesial root was the most important feature for age prediction through both the SVM and RF classifiers. A higher value of the right third molar–mesial root length was a strong predictor of age 19 and above, whereas the lower value of the right third molar mesial root length corresponded to ages 12–18 years. For classification models with 2–Class, 3–Class, and 5–Class, the right third molar mesial root ranked as the most significant feature for age prediction.

In the present study, regression algorithms were also analyzed to evaluate the continuous prediction of age from the length of the teeth. Regression models achieved moderate results on the separated test set. The Extra Tree Regressor achieved the best regression performance of 0.58 R square, 1.81 MAE, and 2.38 RMSE. The present study utilized the dataset from one institute and employed the manual measurement of teeth length. The multi–institute data will be helpful for validation, which is a limitation of the present study. In the future, the development of an automated tool to assess teeth length will enhance rapid processing and may further improve the performance of the predictive models. In this study, among the second and third molars’ mesial and distal roots, the right side of the third molar’s mesial root proved to be a good age predictor.

## 6. Conclusions

The study demonstrated how interpretable Machine Learning and Deep Learning models could be applied to predict age using second and third molar root lengths. The findings of the present investigation showed that the Deep Learning model performed better than the Machine Learning model and the right third molar mesial root length was a good predictor of age. To further improve, diversify, and clinically deploy the algorithms, an extension of the training data set to include more radiographs from multiple sources is required. The findings demonstrate the great prospect of neural network–based Machine Learning and Deep Learning models for assisting dentists in legal response, archaeology, and forensic sciences. Further research is required to extend and include more extensive human–machine comparison investigations. This reproducible approach will aid in the legal, archaeology, and forensic science domains for age estimation.

## Figures and Tables

**Table 1:** Classification performance analysis—LDA feature extractor.

Class Division	Classifier	Accuracy	AUC	Recall	Precision
2–Class	SVM	86.8	0.82	0.93	0.89
	RF	86.0	0.83	0.90	0.90
	Logistic Regression	84.8	0.81	0.90	0.88
3–Class	SVM	66.0	–	0.58	0.50
	RF	60.0		0.69	0.67
	Logistic Regression	60.4		0.69	0.62
5–Class	SVM	44.0	–	0.50	0.50
	RF	42.4		0.42	0.43
	Logistic Regression	40.4		0.51	0.46

LDA: Linear Discriminant Analysis; SVM: Support Vector Machine; AUC: Area under the ROC curve; RF: Random Forest.

**Table 2:** Classification performance analysis—no feature extractor.

Class Division	Classifier	Accuracy	AUC	Recall	Precision
2–Class	SVM	86.4	0.82	0.93	0.88
	RF	85.6	0.80	0.93	0.87
	Logistic Regression	84.0	0.79	0.90	0.87
3–Class	SVM	66.0	–	0.58	0.50
	RF	60.0		0.60	0.65
	Logistic Regression	60.4		0.67	0.62
5–Class	SVM	42.8	–	0.50	0.49
	RF	47.6		0.47	0.44
	Logistic Regression	40.4		0.47	0.44

SVM: Support Vector Machine; AUC: Area under the ROC curve; RF: Random Forest.

**Table 3:** Classification performance analysis—Deep Learning.

Class Division	Model	Accuracy	AUC	Recall	Precision
2–Class	Classification using Deep Learning	87.2	0.88	0.96	0.87

AUC: Area under the ROC curve.

**Table 4:** R square value for regressors.

Regressor	R Square Value	MAE	RMSE
Random Forest Regressor	0.58	1.83	2.40
Extra Tree Regressor	0.58	1.81	2.38
XGBoost Regressor	0.57	1.83	2.41
Gradient Boosting Regressor	0.57	1.85	2.41

XGBoost: extreme gradient boosting.

## Data Availability

All data and material collected are presented in the manuscript. Clarification on any matter can be made through the corresponding author.
